# Supplementary material for: Electrical stimulus artifact cancellation and neural spike detection on large multi-electrode arrays
Source: PLoS Comput Biol. 2017 Nov 13;13(11):e1005842. doi: 10.1371/journal.pcbi.1005842 (PMC5703587; doi:10.1371/journal.pcbi.1005842)
Supplement: S1 Text — (PDF) [file pcbi.1005842.s001.pdf]

Experimental procedures

All electrophysiology data were recorded from primate retinas isolated and mounted on an array of extracellular electrodes as described in previously published literature [1]. Eyes were obtained from terminally anesthetized macaque monkeys (Macaca species, either sex) used for experiments in other labs, in accordance with IACUC guidelines for the care and use of animals. After enucleation, the eyes were hemisected and the vitreous humor was removed. The hemisected eye cups containing the retinas were stored in oxygenated bicarbonate-buffered Ames solution (Sigma) at room temperature during transport (up to 2 hours) back to the lab. Patches of intact retina 3mm in diameter were isolated and placed retinal ganglion cell-side down on a 512-electrode MEA. Throughout the experiments, retinas were superfused with oxygenated bicarbonate-buffered Ames solution at 35°C.

In all experiments the raw voltage signals from each electrode were amplified, filtered, and multiplexed with custom circuitry [2, 3]. Electrodes had diameters of 10-15 μm and were separated by 60 μm. Data were acquired at 20 kHz on all electrodes and bandpass filtered between 43 and 5000 Hz. Charge-balanced, triphasic current pulses with relative amplitudes of 2:-3:1 and phase widths of 50 μs were applied to each electrode, and reported current amplitudes correspond to the charge of the second, cathodal, phase. A platinum ground wire circling the perfusion chamber served as a distant ground in all one-electrode stimulation experiments. In some experiments, a 1 mM tetrodotoxin (TTX) solution in Ames solution was perfused into the retina to inhibit all action potentials in order to directly measure the stimulus artifact in a retinal preparation.

Obtaining the EIs

Retinal ganglion cells (RGCs) were identified in the absence of electrical stimulation using previously described spike sorting techniques [4] and classified into types based on how they respond to a visual white noise stimulus projected onto the retina [5, 6]. For each RGC, thousands of voltage waveforms were averaged on all electrodes, resulting in a spatiotemporal voltage signature specific to that RGC. These signatures are used as templates in our sorting algorithm.

Estimation of mean

Regarding the mean parameter of the artifact kernels, μ, we follow the standard in the applied statistics community: μ is a centering parameter and all the non-random aspects of data should be captured by it. In our case this component is given by what we call the switching artifact, a waveform $A_0 = A_0(e, t)$ that is present regardless of the amplitude of stimulation. We estimate $\hat{\mu}$ by taking the mean of recordings at the lowest amplitude of stimulation (see S1 Fig for details on the characteristics of the switching artifact, and to see the effect of this mean-subtraction stage on recordings).

Dataset details

Real data

Population statistics, data selection

In total, we analyzed 4,045 amplitude series coming from thirteen retinal preparations, giving rise to 1,713,223 trials. These amplitude series are the ones for which reliable human curated data was available. The human analysis of these datasets was required

by various previous research projects (see for example [7–9], where the human analysis procedure is explained). In Table 1 S1 Text we specify details of the thirteen retinal preparations for which human annotation (HA) was available. In some preparations (e.g. 2012-09-24) there is human annotated data from multiple stimulation modalities. Also, in Table 2 S1 Text we specify the population statistics of activation, both in terms of spikes and activation in amplitude series.

For each preparation and stimulus modality, there were characteristic numbers of stimulation patterns and neurons being analyzed. Usually, given a stimulating electrode, human annotation was available for only one, or at most a few neurons (e.g. two or three). However, we considered the totality of EIs of neurons that had strong enough signals (overall EI peak strength greater than 30 μV and $8\mu V$ at at least one stimulating electrode) but restricted performance computations to the subsets of neurons for which human annotation was available.

Bundle detection

Importantly, we restricted our analysis to the stimulation amplitudes that did not lead to gross contamination of recordings due to the activation of entire axonal bundles in the retina (for a recent account of this pervasive phenomenon see [9]), as this would lead to a situation that is not accounted for by our model. For each amplitude series with available human annotation, we determined the maximum amplitude of stimulation that did not lead to activation of a bundle by looking for 'hot' electrodes, distant from the stimulating one, exhibiting high temporal variance in the artifact (here, for simplicity the artifact was estimated by the simple average over traces). Then, we did not consider any amplitude of stimulation beyond the onset of axonal bundle activation, the first amplitude where we identified such hot electrodes. We found that a robust method for estimating this threshold (equivalently, the presence of hot electrodes) was based on a Kolmogorov-Smirnov goodness-of-fit test on the empirical distribution of the (log) temporal variances of the artifact on distant electrodes, with the Gaussianity null hypothesis. The appearance of hot electrodes created a new mode in the distribution, leading to a violation of the normality assumption. We found that by setting the cut-off p-value for this test as 10^{-12} we achieved the best match with axonal bundle activation onsets estimated by human experts.

Refractory period

We considered time windows of $2ms$ ($T = 40$, at a 20khz sampling rate), which is smaller than the usual refractory periods of retinal ganglion cells [10, 11], and which in practice did not lead to multiple neural events for the same neuron on the same trial. Also, spikes were sought in the interval $[0.35, 1.35]$ ms following the onset of the 150 μs triphasic stimulus. This interval encompasses the range were most of the artifact variation occurs; that is, where non trivial artifact cancellation methods are required.

Parallel analysis

For the analysis in Fig 6I we reported times and their variability — the experiment was repeated ten times — for the analysis of the eight single-electrode scans for which for which some human-curated data was available (see Table 1 S1 Text for details on those retinal preparations). These experiments were done on an Intel Xeon E5-2695V2 12C/24T 2.4Ghz 8.0GT/s 30mb CPU, with 20 threads running in parallel.

Preparation ID	Type	#Neurons in preparation	#Neurons with HA	#Trials	#Amplitude series with HA	# Trials per stimulus
2012-09-24-3	S.E.	559	36	400, 805	333	51
2014-09-10-0	S.E.	378	5	40, 802	33	48
2014-11-05-3	S.E.	322	19	37, 940	72	21
2014-11-05-8	S.E.	277	19	37, 644	71	21
2014-11-24-2	S.E.	439	11	36, 078	94	21
2015-04-09-2	S.E.	252	6	31, 775	49	25
2015-04-14-0	S.E.	623	20	86, 655	138	25
2015-05-27-0	S.E.	332	8	30, 368	38	25
Total	S.E.	3,182	124	702, 067	828	n.a.
2012-09-24-3	B.	559	34	187, 612	248	30
2012-09-27-4	B.	482	17	170, 787	184	50
2014-11-24-2	B.	439	9	32, 395	70	30
2015-03-09-0	B.	409	6	67, 332	58	42
2015-04-09-2	B.	252	7	83, 143	79	42
2015-05-27-0	B.	332	8	65, 023	42	50
Total	B.	2,473	81	606, 292	681	n.a.
2014-11-24-2	L.R.	439	14	43, 822	104	21
2015-04-09-2	L.R.	252	4	15, 624	27	25
2015-04-09-3	L.R.	569	2	9, 575	15	25
2015-04-14-0	L.R.	623	25	60, 597	98	25
2015-09-23-2	L.R.	686	28	28, 574	56	25
Total	L.R.	2,569	73	158, 192	300	n.a.
2015-05-27-0	A.	332	4	246, 672	2, 236	10
Total	A.	332	4	246, 672	2, 236	n.a.
Grand Total	All	4443	282	1, 713, 223	4, 045	n.a.

Table 1. Details of the retinal preparations analyzed for each type of stimulation: *Single Electrode* (S.E.), *Bipolar*(B.), *Local Return* (L.R.) and *Arbitrary* (A). stimulation

Simulated data

Simulated data was created by artificially adding neural activity to TTX recordings, in an attempt to faithful mimic the phenomena observed in the real case [1, 12]. Specifically, we considered 83 neurons (the largest subset of the ones targeted in the single-electrode real data analysis so that their EIs did not heavily overlap) and recordings to 380 stimulating electrodes (one at a time) in a TTX experiment with $n_j = 6$ trials to $J = 35$ different stimuli between 0.1 and $3.5\mu A$. Then, given a single stimulating electrode we sampled activation curves for all the neurons whose EI at the stimulating electrode was strong enough, indicating proximity. Activation curves were parametrized by their thresholds, chosen uniformly in the stimulation range, and their steepness, also sampled uniformly. Spikes of those neurons were then sampled from these activation curves with latencies chosen so they would match the human spike sorting results (summarized in S4 Fig) in the following two aspects: 1) they had same

	Trial based		Amplitude series based	
Type of stimulation	#Trials	#Trials with spikes	#Amplitude series	#Amplitude series with activation
Single Electrode	702,067	15,830	828	36
Bipolar	606,292	26,535	681	100
Local Return	158,192	3,564	300	11
Arbitrary	246,672	16,219	2,236	293
All	1,713,223	62,148	4,045	440

Table 2. Population frequency of activation events, for the trial-by-trial and amplitude-series based analysis.

median latency as a function of the distance between the neuron and stimulating electrodes (spiking of nearby neurons has shorter latency) and 2) they had same variance in spike latency as a function of spike probability (in the steady spiking regimes, where the probability of firing is high, latencies are much less variable). Also, to obtain better estimates of false positive rates, we fed the algorithm with 'dummy' neurons (three per amplitude series, with EIs chosen at random from the available set of remaining neurons) with no spiking at all.

All the reported results involving simulations are based on 5000 samples of amplitude series following the above procedure.

References

1. Jepson LH, Hottowy P, Mathieson K, Gunning DE, Dabrowski W, Litke AM, et al. Focal electrical stimulation of major ganglion cell types in the primate retina for the design of visual prostheses. The Journal of Neuroscience. 2013;33(17):7194–7205.

2. Hottowy P, Beggs JM, Chichilnisky EJ, Dabrowski W, Fiutowski T, Gunning DE, et al. 512-electrode MEA system for spatio-temporal distributed stimulation and recording of neural activity. In: Proceedings of the 7th International Meeting on Substrate-Integrated Microelectrode Arrays, Reutlingen, Germany (Stett, A ed), June; 2010. p. 327–330.

3. Hottowy P, Skoczen A, Gunning DE, Kachiguine S, Mathieson K, Sher A, et al. Properties and application of a multichannel integrated circuit for low-artifact, patterned electrical stimulation of neural tissue. Journal of neural engineering. 2012;9(6):066005.

4. Litke A, Bezayiff N, Chichilnisky EJ, Cunningham W, Dabrowski W, Grillo A, et al. What does the eye tell the brain?: Development of a system for the large-scale recording of retinal output activity. IEEE Transactions on Nuclear Science. 2004;51(4):1434–1440.

5. Chichilnisky E. A simple white noise analysis of neuronal light responses. Network: Computation in Neural Systems. 2001;12(2):199–213.

6. Field GD, Sher A, Gauthier JL, Greschner M, Shlens J, Litke AM, et al. Spatial properties and functional organization of small bistratified ganglion cells in primate retina. The Journal of Neuroscience. 2007;27(48):13261–13272.

7. Jepson LH, Hottowy P, Mathieson K, Gunning DE, Dabrowski W, Litke AM, et al. Spatially Patterned Electrical Stimulation to Enhance Resolution of Retinal Prostheses. J Neurosci. 2014;34(14):487–4881.

8. Grosberg LE, Hottowy P, Jepson LH, Ito S, Kellison-Linn F, Sher A, et al. Axon activation with focal epiretinal stimulation in primate retina. Investigative Ophthalmology & Visual Science. 2015;56(7):780–780.

9. Grosberg LE, Ganesan K, Goetz GA, Madugula SS, Bhaskhar N, Fan V, et al. Activation of ganglion cells and axon bundles using epiretinal electrical stimulation. Journal of Neurophysiology. 2017;118(3):1457–1471.

10. Reich DS, Victor JD, Knight BW. The power ratio and the interval map: spiking models and extracellular recordings. Journal of Neuroscience. 1998;18(23):10090–10104.

11. Berry MJ, Meister M. Refractoriness and neural precision. Journal of Neuroscience. 1998;18(6):2200–2211.

12. Sekirnjak C, Hottowy P, Sher A, Dabrowski W, Litke AM, Chichilnisky E. High-resolution electrical stimulation of primate retina for epiretinal implant design. The Journal of neuroscience. 2008;28(17):4446–4456.

13. Saatçi Y. Scalable inference for structured Gaussian process models. University of Cambridge; 2012.

14. Gilboa E, Saatçi Y, Cunningham JP. Scaling multidimensional inference for structured Gaussian processes. Pattern Analysis and Machine Intelligence, IEEE Transactions on. 2015;37(2):424–436.

15. Hashimoto T, Elder CM, Vitek JL. A template subtraction method for stimulus artifact removal in high-frequency deep brain stimulation. Journal of Neuroscience Methods. 2002;113:181–186. doi:10.1016/S0165-0270(01)00491-5.
