# Supplementary material for: Electrical stimulus artifact cancellation and neural spike detection on large multi-electrode arrays
Source: PLoS Comput Biol. 2017 Nov 13;13(11):e1005842. doi: 10.1371/journal.pcbi.1005842 (PMC5703587; doi:10.1371/journal.pcbi.1005842)
Supplement: S2 Text — (PDF) [file pcbi.1005842.s002.pdf]

Some relevant Kronecker product properties

Here we review the main algebraic properties, summarized in [1], that we implement to achieve fast kernel computations. In all of the below, K_d, $(d = 1, \ldots, D)$ are square invertible matrices with dimensions n_d.

Property 0. Associativity. The Kronecker product is associative.

$$(K_1 \otimes K_2) \otimes K_3 = K_1 \otimes (K_2 \otimes K_3)\,.$$

Property 1. Inversion of the Kronecker product. The inverse of a Kronecker product equals the product of their inverses:

$$(K_1 \otimes K_2)^{-1} = K_1^{-1} \otimes K_2^{-1}.$$

Property 2. Kronecker product eigen-decomposition. If

$$K_1 = Q_1 \Lambda_1 Q_1^\top, K_2 = Q_2 \Lambda_2 Q_2^\top,$$

then

$$K_1 \otimes K_2 = Q \Lambda Q^\top$$

where

$$Q = Q_1 \otimes Q_2, \Lambda = \Lambda_1 \otimes \Lambda_2.$$

In other words, the eigen-decomposition of a Kronecker product corresponds to the product of their eigen-decompositions.

Property 3. Trace of a Kronecker product. The trace of a Kronecker product is the product of the individual traces:

$$tr(K_1 \otimes K_2) = tr(K_1) tr(K_2).$$

Property 4. Log determinant of the Kronecker product. The log determinant of the Kronecker product is a weighted sum of the individual log determinants, and the weights are the dimensions:

$$\log |K_1 \otimes K_2| = n_1 \log |K_1| + n_2 \log |K_2|.$$

Property 5. Matrix product between a Kronecker product and a vector. Let v be a $N = \prod_{d=1}^{D} n_d$ dimensional vector, with each n_d of comparable magnitude. Then

$$\bigotimes_{d=1}^{D} K_d v,$$

can be computed efficiently in $O(DN^{(D+1)/D})$ space and time. For implementation details see algorithm 2 in [2], and our code.

References

1. Saatçi Y. Scalable inference for structured Gaussian process models. University of Cambridge; 2012.
2. Gilboa E, Saatçi Y, Cunningham JP. Scaling multidimensional inference for structured Gaussian processes. Pattern Analysis and Machine Intelligence, IEEE Transactions on. 2015;37(2):424–436.
